# Role of minimally invasive glaucoma surgery in the management of chronic open-angle glaucoma

**DOI:** 10.1038/s41598-021-00808-3

**Published:** 2021-11-02

**Authors:** Ojasvi Sharma, Didar Abdulla, Anthony King, Monali Chakrabarti, Tarun Sharma

**Affiliations:** 1 Medical School, University of Nottingham, Nottingham, UK; 2 Ophthalmology Department, Worcestershire Acute Hospitals NHS Trust, Worcester, England UK; 3 Ophthalmology Department, University Hospital, University of Nottingham, Nottingham, UK; 4 Ophthalmology Department, Worcestershire Acute Hospitals NHS Trust, Charles Hastings Way, Worcester, WR5 1DD England, UK

**Keywords:** Diseases, Medical research

## Abstract

To compare the safety and efficacy of phacoemulsification combined with ab-interno trabeculectomy (Trabectome) and phacoemulsification combined with I-Stent inject in patients with medically uncontrolled primary open-angle glaucoma (POAG). A retrospective comparative case series. 70 eyes of 66 patients completed 2 years follow up after these treatments performed in 2017–2018. 35 eyes of 33 patients underwent combined Phaco-Trabectome (PT); and 35 eyes of 33 patients underwent combined Phaco-I-Stent inject (Pi). Patient demographics and preoperative characteristics are comparable. A 20% drop in IOP was achieved in 27 eyes (77.14%) in PT group and 28 eyes (80%) in Pi group (*p* = 0.77). Success rate (target IOP achieved and maintained for 2 years) in advance glaucoma was 25% in PT group and 30.7% in Pi group (*p* = 0.90). In mild to moderate glaucoma, success rate was 85.71% in PT group and 90% in Pi group (*p* = 0.67). There was no significant difference between two groups with regards to mean reduction in glaucoma medications and complication rates. Trabectome and I-Stent combined with phacoemulsification are equally efficacious and safe for treating patients with medically uncontrolled mild and moderate primary open-angle glaucoma (POAG). However, they are not an effective treatment for patients with advanced glaucoma.

## Introduction

Glaucoma accounts for 2% of visual impairment and 8% of global blindness^1–3^. The aim of treatment in glaucoma is to prevent or slow the rate of disease progression by lowering the intraocular pressure (IOP) In patients with medically uncontrolled glaucoma surgical intervention is required to achieve a suitably low IOP^4^. Minimally Invasive Glaucoma Surgery (MIGS) are a recent development in glaucoma surgery which offer IOP reduction and quick post-operative recovery and reduced post-operative follow-up. The MIGS are usually combined with phacoemulsification but can be performed as solo procedures^5^. As these are done via an ab-interno approach they are thus associated with minimal trauma, very little or no scleral dissection and minimal or no conjunctival manipulation^5–7^.

There are some concerns about the efficacy of MIGS which claim they achieve only a modest IOP control^8,9^. However the literature suggests that for each 1 mmHg IOP reduction, disease progression risk is decreased by 11–19%^10,11^. Thus a modest reduction in IOP may be sufficient in patients who do not require a very low IOP or who are unable to tolerate medications or at high risk of complications with more invasive surgery. Therefore, in recent years MIGS have become established as an additional option to the traditional more invasive surgeries within the glaucoma treatment algorithm^5–7^.

Two of the novel minimally invasive glaucoma surgeries (MIGS) that lower IOP are the Trabectome (NeoMedix Corporation, Tustin, CA, USA) or trabeculectomy by an ab-interno approach and I-Stent -trabecular micro-bypass (Glaukos Corp, Laguna Hills, CA, USA). There are several studies establishing their efficacy but there is a paucity of data comparing Trabectome and I-Stent MIGS procedures directly with regard to efficacy and safety^6,7,12–14^. The aim of this study was to compare the safety and efficacy profile of phacoemulsification combined with either ab-interno trabeculectomy (Trabectome) or I-Stent inject in patients with primary open-angle glaucoma (POAG) in a single surgeon / single centre setting.

## Methods

### Study design

A comparative case series of consecutive patients undergoing Phaco- I-Stent (Pi) or Phaco-Trabectome (PT) was undertaken. No formal randomisation process was undertaken but alternative cases were allocated to either PT or Pi. This review received approval from the Worcestershire Acute Hospitals NHS Trust audit and research committee (ID 595/2018) and was consistent with the Tenets of the Declaration of Helsinki. Informed consent was obtained from all subjects. This single surgeon study included POAG patients who underwent combined phacoemulsification and micro-invasive glaucoma surgery (MIGS) with the ab-interno trabeculectomy (Trabectome) or I-Stent devices. All patients who had an IOP above target with worsening glaucoma on maximally tolerated medical treatment and visually significant cataract were consented for combined phacoemulsification with MIGS as a first stage procedure. The target IOP was set prior to surgery for every case at a level expected to prevent further nerve damage.

Exclusion criteria included any form of angle-closure glaucoma, corneal oedema, corneal opacity, increased episcleral venous pressure, prior angle or filtering procedure, history of refractive surgery or ocular trauma or presence of significant health conditions (uncontrolled diabetes, serious cardiovascular problems, bleeding and clotting disorders, and chronic obstructive pulmonary disease). Prior history of laser trabeculoplasty was deemed acceptable.

Patient demographics (age, sex); medical and ocular history including stage and type of glaucoma and the number of ocular hypotensive medications were recorded. Pre-operative examinations included, best-corrected Snellen visual acuity, IOP measured by Goldmann applanation, anterior chamber angle status, vertical cup-to-disc ratio, and automated Humphrey visual field were obtained. Glaucoma staging as per Hodapp classification (recorded as early, moderate, or advanced)^15^ and target IOP were established.

Ocular hypotensive medications were not discontinued before surgery.

### Surgical technique

All patients were operated by a single surgeon using the same surgical protocol under topical anaesthesia. Trabectome surgery was performed before standard phacoemulsification^16^ while I-Stent insertion was done after standard phacoemulsification. Details of I-Stent inject and the surgical techniques have been published^15^. Under gonioscopic view, two I-Stents (inject) were implanted through the trabecular meshwork into Schlemm’s canal, separated by approximately two clock hours. Trabectome surgery was done under gonioscopic view. The electrical ablation was activated to remove approximately a 60 to 120-degree arc of trabecular meshwork and inner wall of Schlemm’s canal while protecting the surrounding tissues. The ablated tissue was aspirated via the irrigation/ aspiration (I/A) lumens of the handpiece.

Postoperative treatment comprised a topical combination of steroids (dexamethasone 0.1% 4 × a day for 4 weeks) and antibiotics (chloramphenicol 0.5% 4 × a day for 2 weeks) following the intervention in each group. In addition, in the Trabectome group pilocarpine 2% eye drops 4 × /day was used for 2 weeks and then were slowly reduced over 6 weeks after surgery to avoid synechiae. Glaucoma medications were continued at preoperative level but were modified over the study period to achieve target IOP in each case but there was no case where glaucoma treatment was completely stopped.

### Follow up

Patients were seen on postoperative day 1, after 1 week and 1, 3, 6, 12, 18 and 24 months. Best-corrected Snellen visual acuity, IOP measurement with Goldman applanation tonometer, number of glaucoma medications, and number of complications were recorded. All patients completed the 2 year follow thus we had data of 100% of eyes at each time point.

### Surgical success

We defined success according to three criteria;

- Patients who achieved an IOP of < 21 mmHg
- Patients showing 20% drop in IOP from baseline
- Patient who achieved and maintained the individualised target IOP (atleast 25% drop in IOP) over 2 years and prevented second stage trabeculectomy.

To compare normally distributed variables, Student’s t-test was performed. Numeric variables that were not normally distributed were compared with the Mann–Whitney U test and the Wilcoxon-Rank-signed-test. Sex, race and incidence of postoperative adverse events were compared between the stent and Trabectome groups using the Fisher exact test. At all times, p-values lower than 0.05 were determined to be significant.

## Results

The study included 70 eyes of 66 consecutive patients. A total of 35 eyes underwent combined Phaco-Trabectome (PT); and 35 eyes underwent combined Phaco-I-Stent inject (Pi). Patient who had bilateral treatment had the same procedure in both eyes. The patient demographics and preoperative characteristics of both operative groups are comparable (*p* > 0.05) (Table 1).

**Table 1:** Demographic comparison between two groups: n = 70 eyes.

Pre-op variables	Phaco-Trabectome (35 eyes/33 subjects)	Phaco-I Stent inject (35 eyes/33 subjects)
Mean age in years	80.7 ± 4.3	80.1 ± 3.8
**Gender**		
Male	16	15
Female	17	18
Mean IOP mmHg	26.6 ± 2.3	25.1 ± 3.1
Mean visual acuity (LogMAR)	0.315	0.312
Mean number of drops	3.2 ± 0.54	3.3 ± 0.4
History of (SLT) selective laser trabeculoplasty	3 eye	2 eyes
**Stage of glaucoma**		
Mild	10	10
Moderate	11	10
Severe	12	13

**Figure 1:**
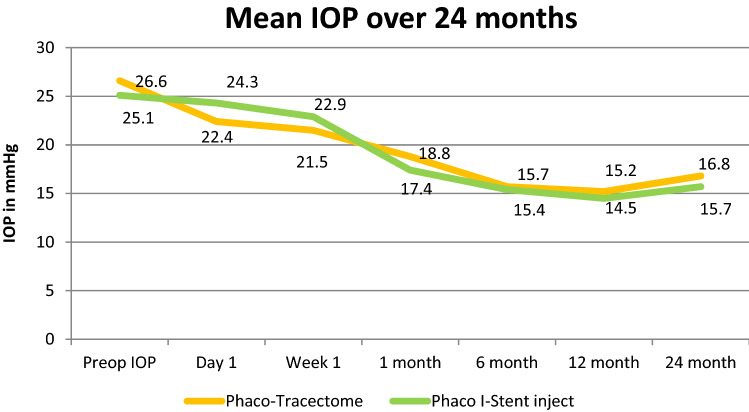
Mean IOP over 24 months (2 years).

The Mean IOP reduced by an average of 7.8 + 1.4 mmHg in the Phaco-Trabectome (PT) group and 7.7 + 1.6 mmHg in the Phaco-I-Stent (Pi) group at the first visit (*p* = 0.89). Mean IOP was at 16.8 + 1.9 mmHg in PT group and 15.7 + 2.1 mmHg in Pi group at 24 months postoperatively (Fig. 1).

### Anti-glaucoma drops

**Figure 2:**
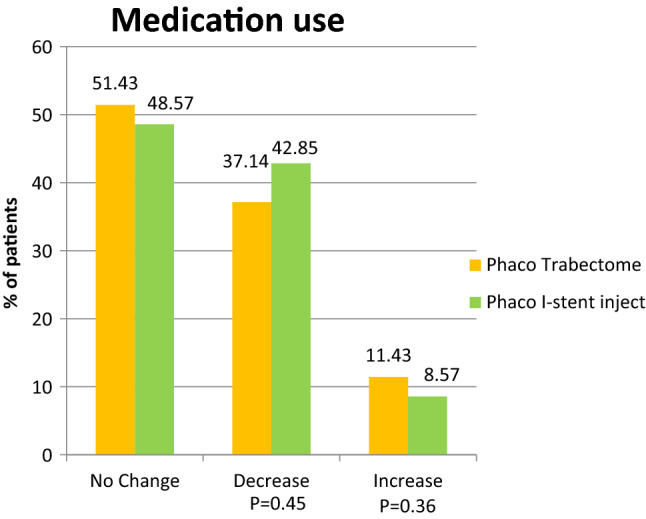
Percentage of patient showing change in Mean number of eye drops at 24 months compared to baseline.

**Figure 3:**
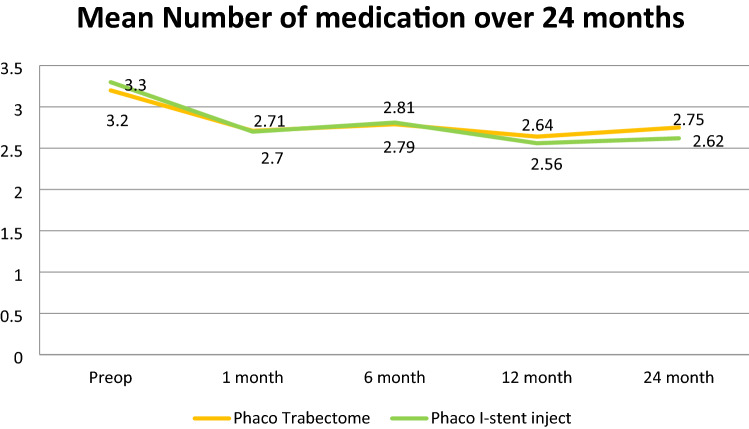
Mean number of drops over 24 months.

In this study, 42.85% of the Pi group had a decrease in drop use at 24 months compared to the 37.14% in the PT group (*p* = 0.45). Following surgery 11.43% in the PT group needed additional glaucoma drops to achieve target IOP compared to 8.57% of the Pi group (*p* = 0.36) (Fig. 2). The PT groups went from an average of 3.2 + 0.54 drops to 2.75 + 0.28 drops post operatively at 24 months. The Pi group averaged 3.3 + 0.43 drops pre-operatively, reducing to an average of 2.62 + 0.38 drops at the 24 months follow up (Fig. 3). The mean drop in number of medications in two groups at 2 years was not significant (*p* = 0.53).

### Surgical success

**Figure 4:**
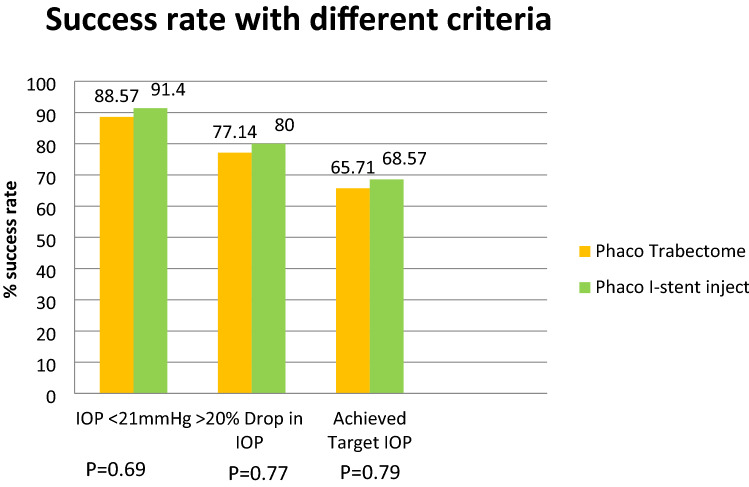
Success rate with different criteria.

Figure 4 shows that there was no material or statistical difference between either groups for any of the 3 success criteria tested.

A total of 31 eyes (88.57%) achieved IOP less than 21 mmHg in PT group while 32 eyes (91.4%) achieved the same in the Pi group (*p* = 0.69). A 20% drop in IOP was observed in 27 eyes (77.14%) in PT group and in 28 eyes (80%) in Pi group (*p* = 0.77). Twenty-three eyes (65.71%) in PT group and 24 eyes (68.57%) in Pi group achieved and maintained the target IOP and avoided second stage trabeculectomy over a 2-year period (*p* = 0.79). Twelve eyes in PT group and elevan eyes in Pi group failed to achieve their individualised target IOP therefore required a trabeculectomy. Amongst those eyes which required trabeculectomy 9 out of 12 eyes in PT group and 9 of 11 eyes had advance glaucoma while 3 eyes in PT group and 2 eyes in Pi group had moderate glaucoma. Success rate (target IOP achieved and maintained for 2 years) in advance glaucoma was 25% (3 out of 12 eyes) in PT group and 30.7% (3 out of 11 eyes) in Pi group (*p* = 0.90) but was much higher in mild to moderate glaucoma was 85.71% (18 out of 21 eyes) in PT group and 90% (18 out of 20 eyes) in Pi group (*p* = 0.67).

### Visual acuity

**Figure 5:**
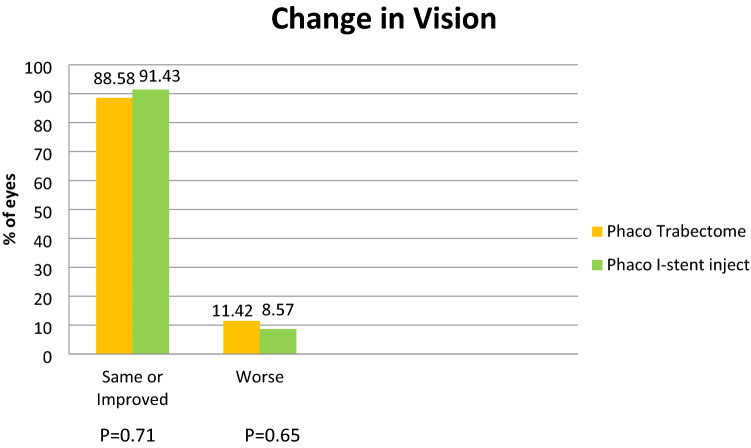
Change in vision.

Visual acuity improved or remained at the same level in 88.58% in PT vs 91.43% in Pi group. We saw 11.42% (4/35) of the patients in the PT group and 8.57% (3/35) in the Pi group resulted in poorer acuities (at least 1 or more line drop in Snellen visual acuity) at the 24-month period (Fig. 5). 2 eyes in each group showed worsening of age related macular degeneration. The cause of poor vision in remaining 2 eyes in PT group and 1 eye in Pi group was cystoid macular oedema related changes.

### Complications

Complication rates in both groups were low, however in the Pi group, 2 eyes developed cystoid macular oedema compared to 3 eyes in the PT group. OCT of macula was done if best corrected visual acuity was less than 6/6. All cases of CMO resolved except for one case in the PT group. There were no cases of hypotony where IOP dropped below 8 mmHg at any stage. Two eyes in each group developed wet AMD causing poorer visual acuity.

## Discussion

Glaucoma leads to blindness in a significant number of individuals, even while patients are receiving therapy^2,3^. Hattenhauer et al.^17^ estimated a probability of 9% for bilateral blindness and a 27% probability of blindness in at least one eye from classic glaucoma and treated ocular hypertension at 20 years. Early trabeculectomy provides significantly better protection of visual field in the long term^18^. However due to concerns regarding potential sight threatening complications, trabeculectomy is often only offered in advanced stages and remains the effective IOP lowering surgical procedure^3^. The focus of research in last two decades has been in developing new therapeutic strategies which aim to increase the efficacy of glaucoma treatment and improve the quality of life with minimal complications^19,20^.

MIGS can be used to further manage medically uncontrolled glaucoma and may provide sufficient control to avoid more invasive glaucoma surgery. Ab-interno trabeculectomy (Trabectome) and I-Stent inject have been shown to be effective in lowering the IOP with minimal side effects^5–9,19,21–25^. The evidence on the efficacy of MIGS compared to other therapies is still limited and is based on few RCTs of acceptable quality, a larger number of Non-Randomised Studies and uncontrolled before/after case series^13,14^. Lavia et al.^5^ conducted a meta-analysis and showed a decrease of IOP and a reduction of glaucoma medications after MIGS surgery with a low complication rate.

In this study the drop in mean IOP was similar in the two groups at all stages. In the immediate postoperative period, PT group was using additional pilocarpine 2% eye drops to prevent formation of synaechiae. As pilocarpine was completely stopped in all cases at 6 weeks, it should not have any impact on the longer term result. Our intention is to compare the mid to longer term effect of these two procedures. The drop in mean IOP of 9.4 + 2.2 in PT group and 9.8 + 3.4 mmHg Pi group was seen after 2 years. The reduction in the number of eye drops was 0.55 + 0.15 in PT group and 0.58 + 0.18 in the Pi group. This suggests that both procedures are reasonably effective in reducing IOP. Studies comparing Trabectome vs I-Stent showed results in favour of one method or the other. Gonnermann et al.^7^ found a 34% reduction in IOP in the I-Stent group compared to 30% drop in the Trabectome group but results were not statistically significant. The reduction in mean number of medications at 12 months was higher in I-stent group and was statistically significant. Kurji et al.^6^ reported greater drop in absolute IOP in the Trabectome group but results were not statistically significant. The meta-analysis of these three studies on IOP change showed no difference between Trabectome and I-Stent as combined procedures^5^. Khan et al.^12^ showed statistically significant greater reduction of IOP and medications in the I-Stent group than the Trabectome group. Two recent studies showed greater IOP reduction with fewer medications after Phaco Trabectome surgery than Phaco I-stent surgery^13,14^.

Our results were similar to these studies with no significant difference in IOP change between PT and Pi group. Gonnermann et al.^7^ did not find any significant complications in either group. Khan et al.^12^ and Kurji et al.^6^, both reported significantly more early postoperative complications in Trabectome group but these resolved within a week. After 1 week there was no statistically significant difference between Trabectome or I-Stent group in both the studies. Our complication rates were lower like all the three studies with no significant difference between the two groups. We however found no significant differences between interventions for success.

Two thirds of patients in our study (65.71% in PT group and 68.57% in Pi group) achieved their predetermined target IOP and maintained it throughout the study period (2 years) and avoided second stage trabeculectomy (*p* = 0.79). The subgroup analysis showed success rate in advance glaucoma was 25% in PT group and 30.7% in Pi group (*p* = 0.90) but was much higher in mild to moderate glaucoma was 85.71% (18/21) in PT group and 90% (18/20) in Pi group (*p* = 0.67). This suggests that these interventions should not be considered in cases of advanced visual field loss.

Our study showed slightly higher incidence of cystoid macular oedema (CMO): 8.5% (3 Eyes) in PT group vs 5.7% (2 Eyes) in Pi group (higher than surgeon’s CMO rate in routine phacoemulsification (1.2%). CMO resolved in both groups except for one case in PT group where it persisted even after treatment and caused reduced visual acuity. A total of 4 eyes in PT group and 3 eyes in Pi showed worsening of visual acuity by at least one line on Snellen chart. Two eyes in each group had reduced vision due to wet AMD. It means that at least one eye in each group suffered irreversible changes to the macula with CMO even after resolution of CMO.

Most of the studies claimed success in MIGS in mild to moderate glaucoma^5,8,9^. Some recent studies claim that MIGS is very effective in advance glaucoma as well^23,25,26^. In our study 75% (9 out of 12 eyes) in PT group and 69.3% (8 out of 11 eyes) in Pi group required trabeculectomy within 2 years. This suggests that in patients with more advanced glaucoma only 25%-30% achieve the desired low target IOP with these two techniques. Therefore alternative of a trabeculectomy should also be discussed with these patients as this is more likely to be successful in this group of patients of achieving a low IOP required. Once the pros and cons of both procedures have been discussed the patients should then make the final choice as to which surgery they would prefer.

Our results show a decrease of IOP and a reduction of glaucoma medications after MIGS surgery with a low complication rate. The reduced operating time and less frequent follow up and low complication rate compared to trabeculectomy may avoid the need for trabeculectomy in many patients who do not require a very low IOP^27^. This may prove cost effective and these procedures may help health care providers cope with an ever-increasing demand due to an aging population. There are cost implications for both MIGS procedure. Trabectome requires a huge upfront cost (> £35,000) plus a regular cost of head pieces (variable depending upon the size of the order) while cost for I-stent is almost £1000 per case. These cost implications need to be taken in to account when establishing cost effectiveness of these procedures.

MIGS may help a significant number of POAG patients to reduce their glaucoma medication burden and maintain their quality of life by slowing / stabilising the disease progression with significantly less risks taken.

One of the weaknesses in our study is that we do not have a comparable group of patients undergoing phacoemulsification alone without the additional MIGS procedures. The reported reduction of IOP following cataract surgery is of approximately 5 mmHg. In both of our groups mean drop in IOP in mmHg after 2 years (9.8 + 3.4 in PT and 9.4 + 2.2 in Pi) was almost double of the mean drop of IOP following cataract surgery alone (5 mmHg) with slight reduction in mean number of medications used. This is highly suggestive of a positive impact of these procedures over and above that of cataract surgery alone.

The limitations of this report include the retrospective nature of the data collection, lack of randomisation and potential bias in choosing one technique over another. Advantages include lack of attrition and 2 years follow-up.

## Data Availability

The datasets generated during and/or analysed during the current study are available from the corresponding author on reasonable request.
